# Combining the responses of habitat suitability and connectivity to climate change for an East Asian endemic frog

**DOI:** 10.1186/s12983-021-00398-w

**Published:** 2021-03-26

**Authors:** Zhenhua Luo, Xiaoyi Wang, Shaofa Yang, Xinlan Cheng, Yang Liu, Junhua Hu

**Affiliations:** 1 Institute of Evolution and Ecology, School of Life Sciences, Central China Normal University, Wuhan, 430079 China; 2 Chengdu Institute of Biology, Chinese Academy of Sciences, No. 9 Section 4, Renmin Nan Road, Chengdu, 610041 China; 3 State Key Laboratory of Biocontrol, School of Ecology, Sun Yat-sen University, Guangzhou, 510275 China

**Keywords:** Climatic condition, Giant spiny frog, Habitat connectivity, Dispersal ability, Suitable habitat

## Abstract

**Background:**

Understanding the impacts of past and contemporary climate change on biodiversity is critical for effective conservation. Amphibians have weak dispersal abilities, putting them at risk of habitat fragmentation and loss. Both climate change and anthropogenic disturbances exacerbate these risks, increasing the likelihood of additional amphibian extinctions in the near future. The giant spiny frog (*Quasipaa spinosa*), an endemic species to East Asia, has faced a dramatic population decline over the last few decades. Using the giant spiny frog as an indicator to explore how past and future climate changes affect landscape connectivity, we characterized the shifts in the suitable habitat and habitat connectivity of the frog.

**Results:**

We found a clear northward shift and a reduction in the extent of suitable habitat during the Last Glacial Maximum for giant spiny frogs; since that time, there has been an expansion of the available habitat. Our modelling showed that “overwarm” climatic conditions would most likely cause a decrease in the available habitat and an increase in the magnitude of population fragmentation in the future. We found that the habitat connectivity of the studied frogs will decrease by 50–75% under future climate change. Our results strengthen the notion that the mountains in southern China and the Sino-Vietnamese transboundary regions can act as critical refugia and priority areas of conservation planning going forward.

**Conclusions:**

Given that amphibians are highly sensitive to environmental changes, our findings highlight that the responses of habitat suitability and connectivity to climate change can be critical considerations in future conservation measures for species with weak dispersal abilities and should not be neglected, as they all too often are.

**Supplementary Information:**

The online version contains supplementary material available at 10.1186/s12983-021-00398-w.

## Background

Changing climatic conditions have begun altering or removing the conditions that are necessary for many species to exist on the Earth’s surface, leaving a clear fingerprint on global biodiversity [1–3]. Climate change induces shifts in the geographic ranges, abundance, phenology, individual behaviour or physiology, and genetic diversity of natural populations [4–8]. Furthermore, the rapid warming rates projected for the planet [1, 9] are expected to doom many species to extinction because climatically suitable habitats may disappear or become inaccessible due to geographic barriers or conditions that make it unsuitable for species to disperse [2, 3]. Regardless of other factors, the impacts of climate change on species are becoming increasingly apparent [5, 10]. It is clear that immense challenges to conservation and environmental management in the face of climate change exist [7, 11, 12].

Habitat degradation, fragmentation and loss are the primary causes of biodiversity loss in most of the world’s ecosystems [13]. Habitat fragmentation/isolation impedes dispersal, increasing inbreeding, genetic drift, and, hence, the likelihood of local extirpation due to demographic stochasticity [14, 15]. These factors might limit species’ abilities to adapt to environmental changes and can thus cause extinctions [13]. Habitat connectivity, the degree to which a landscape facilitates or impedes dispersal among habitat patches, confers ecosystems with greater resilience towards disturbances and thereby facilitates population viability [16]. As a consequence of the effects of climate change and human activities, habitat connectivity is often degraded, causing negative impacts to global biodiversity [12, 17].

Amphibians are highly sensitive to environmental changes due to their low mobility and strict physiological constraints [2, 18]. They are at disproportionately high risk of extinction, with many species’ populations rapidly declining. Amphibians can thus act as useful indicators of environmental changes across space and time [2, 18, 19]. One amphibian species that is facing a dramatic population decline is the giant spiny frog (*Quasipaa spinosa*), an endemic species to East Asia [20, 21]. Over-harvesting, observed distribution shrinkage, and ongoing habitat destruction and degradation have caused wild populations of *Q. spinosa* to significantly decrease, with an estimated decline of more than 30% over the last 15 years (roughly three *Q. spinosa* generations) [20, 22]. Their main range falls within a densely populated region of China, where, despite a long history of human occupation, recent developments have put new pressure on the species. There are currently no protective regulations for *Q. spinosa* except for collection prohibitions within nature reserves since the frog is not yet listed as a National Protected Wild Animal Species under Chinese law. All these factors create an urgent need to better understand the interactive threats of climate change and fragmentation to this species.

Here, we inferred the impacts of historical and future climate changes on the landscape connectivity of habitats of *Q. spinosa*. We simulated the environmental suitability for this species over time and measured its potential distribution as a function of the varying climatic conditions from the last interglacial period (LIG, 120–140 kyr BP) to the projected future climate, i.e., for the years 2050 and 2070. Then, we characterized landscape connectivity among habitat patches and determined how climate change could affect the dispersal of individuals. Overall, these efforts can provide novel insight into how habitat connectivity contributes to conserving amphibian species in a changing world.

## Methods

### Study species

*Quasipaa spinosa* is a large-sized anuran. Adult males are characterized by keratinized spines on their chests that appear during breeding seasons [22]. The distribution of *Q. spinosa* covers 12 provincial-level regions across central, southern and southwestern China (including Yunnan, Guizhou, Guangxi, Guangdong, Hong Kong, Fujian, Jiangxi, Hunan, Hubei, Anhui, Jiangsu and Zhejiang) and northern Vietnam [20, 22]. It inhabits rocky streams in evergreen forests and open countrysides/fields on hills and mountains. Its elevation range is 200–1500 m a.s.l. [20, 22, 23]. This species is considered an important food and medicinal resource according to traditional views [22]. It has been collected for consumption throughout its range for many decades, a practice that persists. To make matters worse, habitats of *Q. spinosa* have been degraded and/or destroyed by agricultural pollution and the construction of dams for hydropower projects [20, 22, 24]. This species is currently classified as “vulnerable (A2abc)” on the IUCN Red List [20]. Despite the initiation of captive breeding programmes in the 1980s, low fertilization and hatching rates, diseases, and high overwinter mortality mean that the cultivation of *Q. spinosa* strongly depends on the supply of tadpoles or adults from wild populations [24]. Thus, other conservation measures to arrest the population decline of this species are urgently required.

### Data collection and processing

**Fig. 1:**
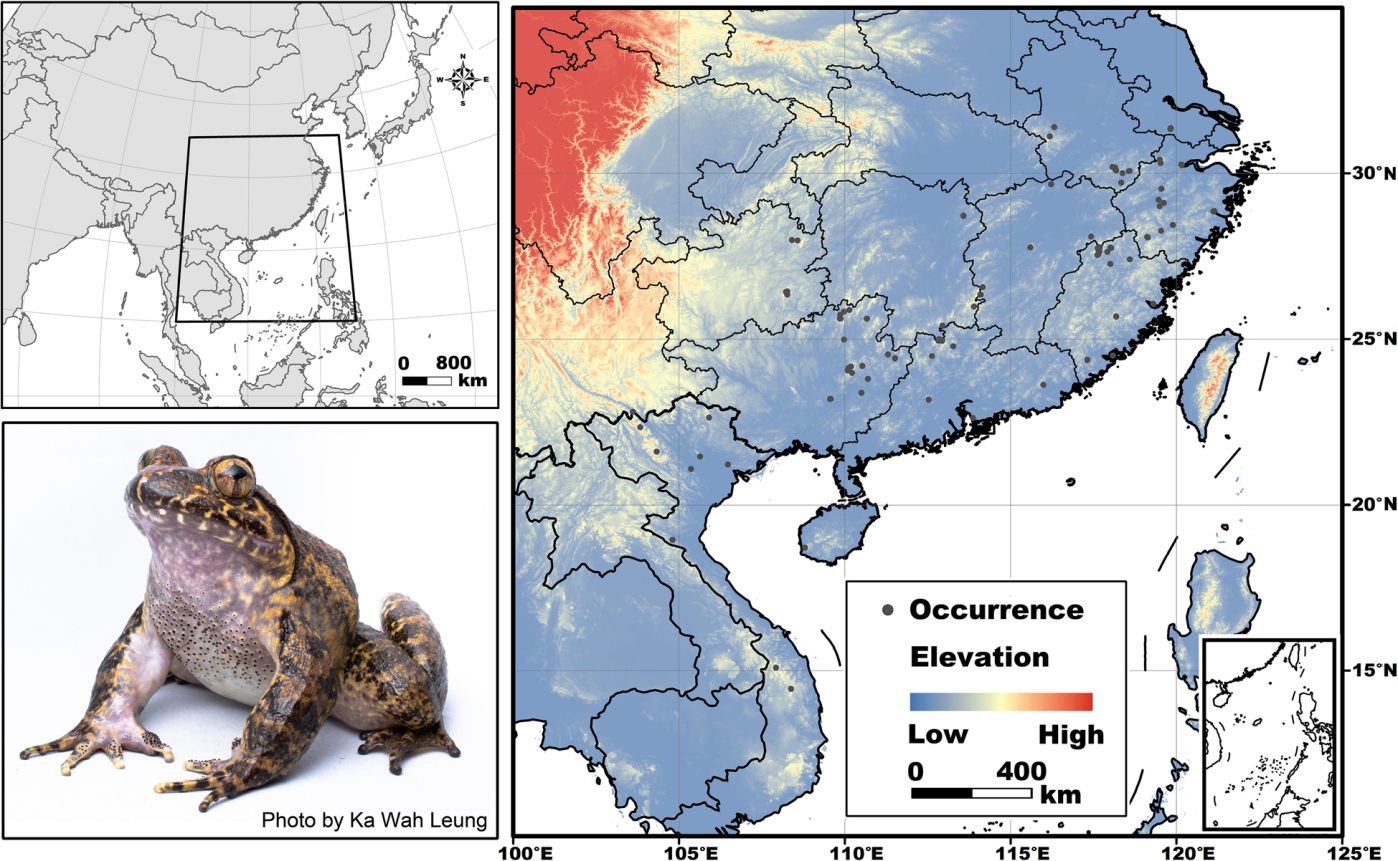
Study area and occurrence records of *Quasipaa spinosa*

The study area constituted central and southern China and adjacent countries in Southeast Asia, including Vietnam, Laos, Thailand, Cambodia, the Philippines, and Myanmar (10–35°N, 100–125°E; Fig. 1). We collected a total of 1714 occurrence records of *Q. spinosa* from field expeditions conducted by Chengdu Institute of Biology (CIB) personnel (2010–2015), supplemented with location data from the literature [24–26], the Global Biodiversity Information Facility (GBIF; http://www.gbif.org/), and georeferenced specimen records in the Herpetological Museum of the Chengdu Institute of Biology (CIB). To avoid georeferencing errors and over-fitting in the following modelling, we checked all location data in ArcGIS 9.2 (ESRI, Redland, USA) and removed duplicate occurrences at a spatial resolution of 1 × 1-km so that each grid cell had only a single record (in each grid cell, we randomly selected one occurrence and deleted all the other occurrences) [27]. We obtained a total of 136 occurrences composing the final dataset (Fig. 1).

Based on the habitats of *Q. spinosa* [20, 22] and our field experiences, we considered five types (i.e., climate, habitat, biogeography, topography, and human impact) of environmental variables, including 22 parameters (Table S1 in Additional File 1 [28–33];) that may impact the habitat use and distribution of *Q. spinosa* in our modelling processes. For several variables of each type, we can use dimension-reduction techniques (e.g., correlation analysis, clustering algorithms) by pruning the variables with possible redundancy [7, 34]. This procedure was to minimize the risk of overfitting species-environment relationships. Specifically, we retained those variables that gave a higher value in the jackknife analysis [35], which consequently yielded the 14 climatic variables and eight other types of variables (Table S1 in Additional File 1). All variables were transformed into 1 × 1-km equal-area grid rasters in ArcGIS 9.2 and projected onto the UTM WGS 1984 projection.

To capture the uncertainties in future climate projections [9] and represent different temperature sensitivities and greenhouse gas emissions, we considered six global climate models (GCMs: NCC-NorEsm1-M, MRI-CGCM3, BCC-CSM1.1, CSIRO_MK_3_6_0, MIROC_ESM_CHEM, and IPSL_CM5A_LR) for four representative greenhouse gas concentration pathways (RCPs: RCP2.6, RCP4.5, RCP6.0, and RCP8.5) from the IPCC 5th Assessment report [1]. We obtained the 14 climatic variables for 2050 and 2070 from the WorldClim 1.4 dataset [36], that is, we collected 24 projections (6 GCMs × 4 RCPs) for each variable at each time slice to illustrate the temperature and precipitation conditions at that time. For the past climate, variable layers were obtained for the time frames of the Mid-Holocene (MidH, ~ 6 kyr BP) [36], the Last Glacial Maximum (LGM, ~ 22 kyr BP) [37], and the LIG [38]. Three GCMs (CCSM4, Mirco-ESM, and MPI-ESM-P) were used for both the MidH and LGM, but only one GCM (CCSM) was available for the LIG.

For each climate change scenario, climatic variables were used to make projections into the past and the future. The non-climatic variables (i.e., AI, aridity index; PET, annual potential evapo-transpiration; Landcover, land cover type; NPP, net primary productivity; Biome, terrestrial ecoregion; HFP, human footprint index) were assumed to be constant over time, as past and future estimates of these factors were, unfortunately, not available. In addition, these variables are likely to be determined not only by climatic drivers but also a wide range of socioeconomic drivers, and any simple estimates or extrapolations could be misleading [cf. 39]. Thus, their constancy is an assumption that presents a conservative prognosis and limits additional uncertainties [cf. 39]. To determine the influence of the non-climatic data, we predicted and analysed the environmental suitability for *Q. spinosa* by comparing two competing models [39–41]: the full model (using all 22 variables) and a climate-only model (using only the 14 bioclimatic variables).

### Ensemble distribution modelling and climate change impacts

We predicted the potential distributions of *Q. spinosa* under current climatic conditions using a maximum entropy approach in MaxEnt v3.3.3 k [42, 43]. MaxEnt has been shown to have good performance and consistently outperforms many other methods, especially with small sample sizes, noisy input data, and correlated parameters [43, 44]. As an inappropriate model complexity (e.g., inappropriate combination of feature classes, level of regularization, or variable selection) or data organization (e.g., occurrence and background localities partition) can typically reduce the accuracy of inferred habitat quality, we selected model settings and conducted model choices using the ENMeval 0.1.0 package in R 4.0.3 software [45–48]. Following the method outlined in Muscarella et al. [47], five feature class values (FC: linear, quadratic, product, threshold, hinge), eight regularization multiplier values (RM: 0.5, 1, 1.5, 2, 2.5, 3, 3.5, 4), five locality partition methods (jackknife, randomkfold, block, checkerboard1, checkerboard2) were calculated; the mean values of the area under the receiver operating characteristics curve using the testing data across all data bins (mean AUC_test_) and the mean values of the difference between the training and testing AUCs across all data bins (mean AUC_diff_) were computed as the evaluation metrics for the goodness-of-fit and the degree of overfitting of the model. For model selection, we used the Akaike Information Criteria corrected for a small sample size (AICc) to compare different variable combinations [46, 49]. The existence of sampling bias in background points could decrease modelling effectiveness; thus, we used a kernel density estimator (KDE) surface to create 10,000 random background points in the Software for Automated Habitat Modelling (SAHM) [50]. As the models incorporating all environmental factors (i.e., 22 variables for the full model, 14 variables for the climate-only model) and with the conditions FC = linear, RM = 1, and the randomkfold (k = 10) partition method showed the best performances (see Results), 80% of the localities were randomly selected to generate a training set, and the remaining 20% of the localities were withheld to use as an evaluation set. Other model settings were adopted, including the maximum number of iterations (500) and the convergence threshold (10^− 5^) [43]. We selected the logistic output format with suitability values ranging from 0 to 1 and conducted jackknife procedures to evaluate the relative contribution of each variable [43].

We then projected the contemporary species-environment relationships into different time slices (the past: LIG, LGM and MidH; the future: 2050 and 2070). For each time slice and each environmental variable group, we ran ten cross-validation replications of the model and weighted them by their AUCs to obtain an ensemble distribution prediction [51, 52]. Next, we averaged the outputs for each past (3GCMs each for LGM and MidH) and future time slice (6 GCMs × 4 RCPs) to achieve a suitability map for each timeframe.

To convert continuous outputs into presence-absence maps, we extracted the suitability values of the occurrences of *Q. spinosa* from the raw modelling results and calculated the mean values as the thresholds (full model: 0.61, climate-only model: 0.58) [53]. That is, areas with predicted suitability values above and below the threshold were considered “present” (suitable) and “absent” (unsuitable), respectively. Considering the home ranges, movements and dispersal abilities of anurans [54], we excluded habitat patches < 10 km^2^ in size from the subsequent analyses. To quantify the impacts of climate change, we first calculated the total area of suitable habitats, the number of suitable habitat patches, and the area of the largest and smallest habitat patches for each time slice. Then, the latitudes, longitudes, and elevations of the “present” grid cells were extracted, and one-way ANOVAs were conducted to detect their variations among time slices. In addition, to show the effectiveness of the existing protected area network on protecting *Q. spinosa* habitats, we overlaid the distributions of protected areas ([55]; only national and international protected areas were included as they have relatively reliable management efforts [56, 57]) and suitable habitat patches at present and in the future (for the current time, 2050, and 2070; for the full model and the climate-only model). We calculated the percentages of the area and the percentages of the number of patches of suitable habitats covered by the protected areas for each time slice.

### Landscape connectivity assessment

Based on the response curves, which show the relationships among environmental variable gradients and the predicted suitability of species, we ranked each variable into rasters ranging from 1 to 10, with higher values indicating higher habitat quality (Table S2 in Additional File 1). Low-quality grid cells have high movement costs for organisms, whereas good habitats are associated with lower resistance to movement [14, 58]. We then reversed the integer categories of each variable for habitat quality to reclassify the movement cost score of *Q. spinosa* by grid cell (Table S2 in Additional File 1). To create a movement cost surface for each time period, we weighted the reclassified rasters of environmental variables with training gains with only the variables obtained from the jackknife procedure (Table S2 in Additional File 1), and then averaged the movement cost surfaces from all the GCMs and RCPs (3 GCMs for LGM and MidH, 6 GCMs × 4 RCPs for 2050 and 2070).

Landscape connectivity was assessed using a pairwise cost distance method, the Cost Distance Tool in ArcGIS 9.2 [59]. This method evaluates the best potential dispersal linkage by summing the movement cost of each grid cell between two core habitat patches. The best potential dispersal linkage is the one that has the least resistance (the least cost patch-to-patch path, LCP). Based on the movement cost surfaces and suitable habitats, we created pairwise LCPs among habitat patches for each time slice with the Corridor Design Tool [59]. We accounted for the potential limits to dispersal distances by using four LCP length caps: 20 km, 50 km, 100 km, and no limit. To investigate the dynamics of landscape connectivity as consequences of climate change over time, we compared the numbers, mean lengths, and mean movement costs of the LCPs among the time slices.

## Results

The full model with 22 environmental variables (mean ± SE: AUC_test_ = 0.948 ± 0.033, AUC_diff_ = 0.023 ± 0.002, AICc = 712.328, AICw = 0.412) and the climate-only model with 14 variables (AUC_test_ = 0.922 ± 0.044, AUC_diff_ = 0.029 ± 0.003, AICc = 3005.074, AICw = 0.407) both had great performances (FC = linear, RM = 1, randomkfold partition method: k = 10). Precipitation in the dry season (Bio14 & Bio17), annual precipitation (Bio12) and precipitation seasonality (Bio15) were the most important contributing parameters for model development, and the aridity index (AI) and ecoregion type (Biome) had obvious impacts on predicted suitability in the full model (Fig. S1 in Additional File 2).

**Fig. 2:**
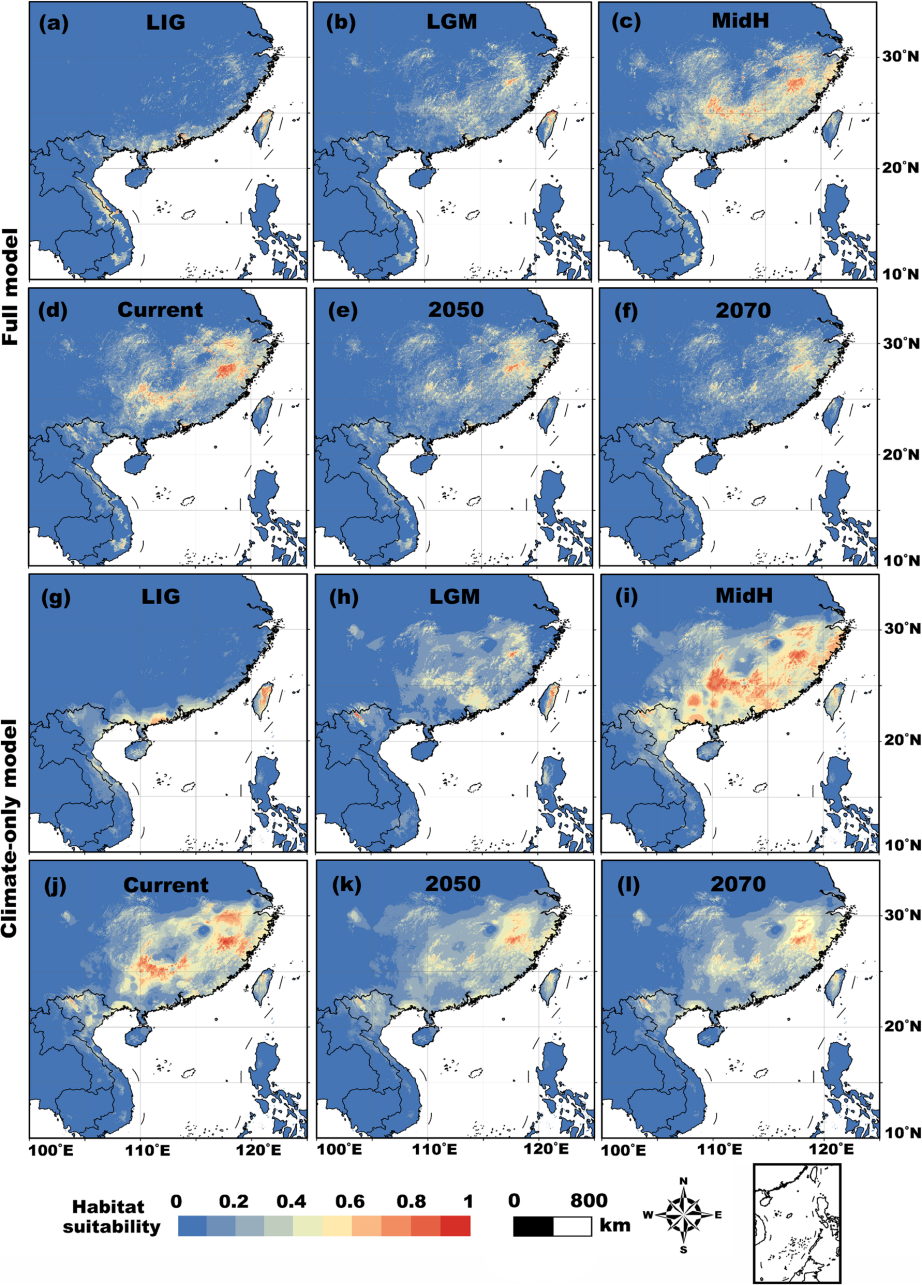
Habitat suitability predictions for *Quasipaa spinosa*. Top two rows: outputs are from the full model (all 22 environmental variables are included). Bottom two rows: outputs are based on the climate-only model (only the 14 bioclimatic variables are included)

**Fig. 3:**
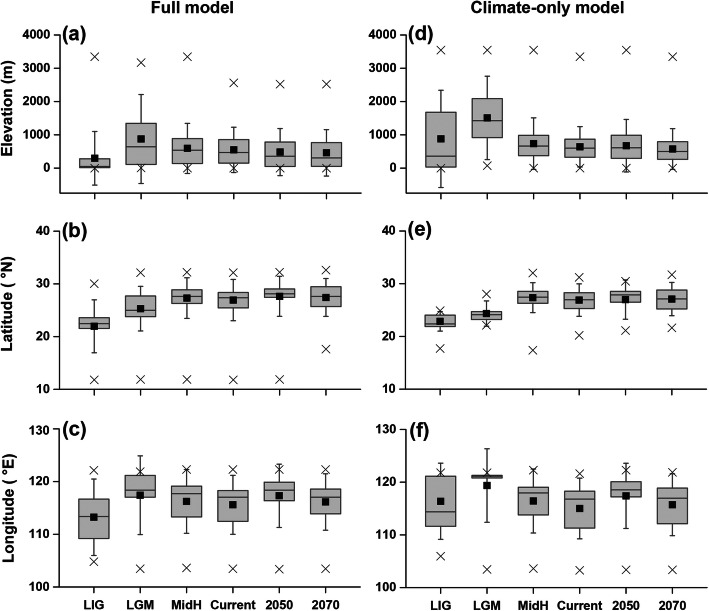
Distribution shifts of *Quasipaa spinosa* at the elevational, latitudinal, and longitudinal dimensions under climate change (over the time periods of the LIG, the LGM, the MidH, Current, 2050, and 2070). **abc**: full model (all 22 environmental variables are included), df = 5, 218,228, α = 0.05; **def**: climate-only model (only the 14 bioclimatic variables are included), df = 5, 589,914, α = 0.05. Black squares show the means, solid lines show the medians, the edges of boxes are the quartiles, the crosses are the lower and upper adjacent limits, and the whiskers are the standard deviations

**Fig. 4:**
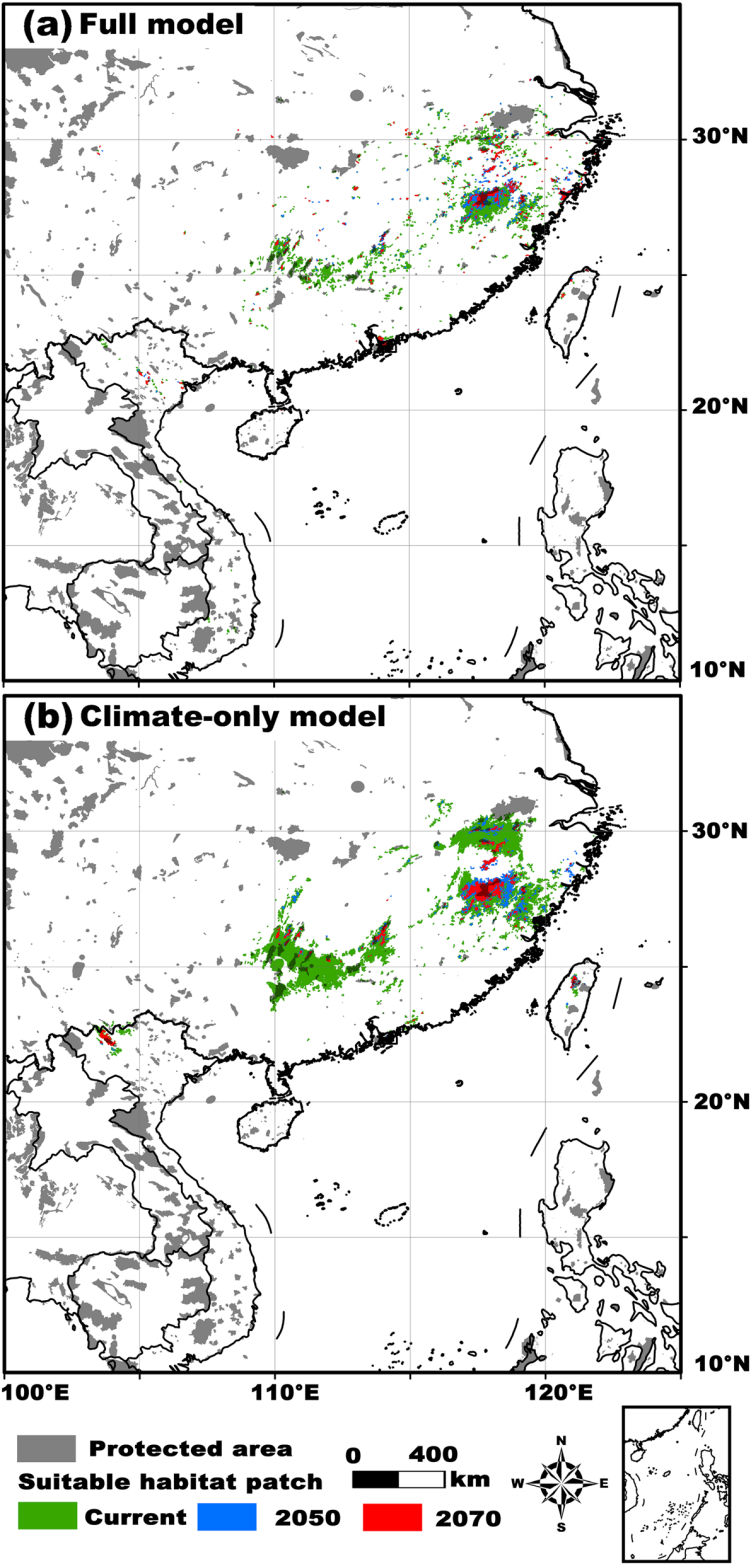
Overlaps between the existing protected area network and the patches of suitable habitats for *Quasipaa spinosa*

Over time, the suitable habitats of *Q. spinosa* moved noticeably as a consequence of climatic variations among the different time slices. Relatively wider ranges of suitable habitats were shown in the MidH and current time periods than in the other time slices, with regions of higher suitability concentrated in the central-south and south-eastern parts of China and in the eastern part of the Indo-Chinese Peninsula. More restricted highly suitable ranges were revealed in the Quaternary LIG and LGM and in the future (Fig. 2). The total area, number, and mean size of habitat patches decreased from the LIG to the LGM, followed by obvious increases in the MidH and the present before declining under projected future conditions (i.e., in 2050 and 2070; Table 1). Compared with the full model, the outputs projected by the climate-only model were similar but showed larger areas of high suitability. Overall, we found a significant latitudinal increase in the area of suitable habitat over time (ANOVA: full model, *P* = 0.01; climate-only model, *P* = 0.02; Fig. 3), indicating northward range expansion across the climate change scenarios. No significant shift along either the elevational or the longitudinal gradient was detected (all *P* > 0.05; except for a slightly upward trend for the full model, *P* = 0.04; Fig. 3). Our results showed that, based on the full model (the climate-only model), the existing protected area network covered only 12.1% (10.3%) of the total *Q. spinosa* habitat area, and only 12.2% (14.3%) of the suitable habitat patches overlapped or partly overlapped with protected areas at the present (Fig. 4). For 2050, the habitat area and patch number percentages were 29.2 and 12.5% based on the full model and 22.7 and 21.6% based on the climate-only model, respectively (Fig. 4). For 2070, the habitat area and patch number percentages were 31.8 and 15.6% based on the full model and 30.1 and 30.0% based on the climate-only model, respectively (Fig. 4).

**Table 1:** Total suitable habitat areas, numbers of habitat patches, and mean areas of habitat patches for *Quasipaa spinosa* over time

Time period	Total area (km^2^)	No. of patches	Mean area (mean ± SD, km^2^)
Full model			
LIG	12,466.4	226	60.2 ± 147.2
LGM	10,212.9	185	51.8 ± 227.9
MidH	87,121.6	1074	81.1 ± 584.7
Current	74,222.3	829	89.5 ± 760.8
2050	22,900.1	393	58.3 ± 348.2
2070	11,313.7	269	42.1 ± 141.6
Climate-only model			
LIG	24,822.7	47	528.1 ± 2257.4
LGM	5850.0	45	130 ± 309.3
MidH	337,334.8	734	459.6 ± 3996.2
Current	174,089.0	505	344.7 ± 2998.8
2050	32,376.3	213	152 ± 964.9
2070	15,449.0	130	118.8 ± 602.2

**Fig. 5:**
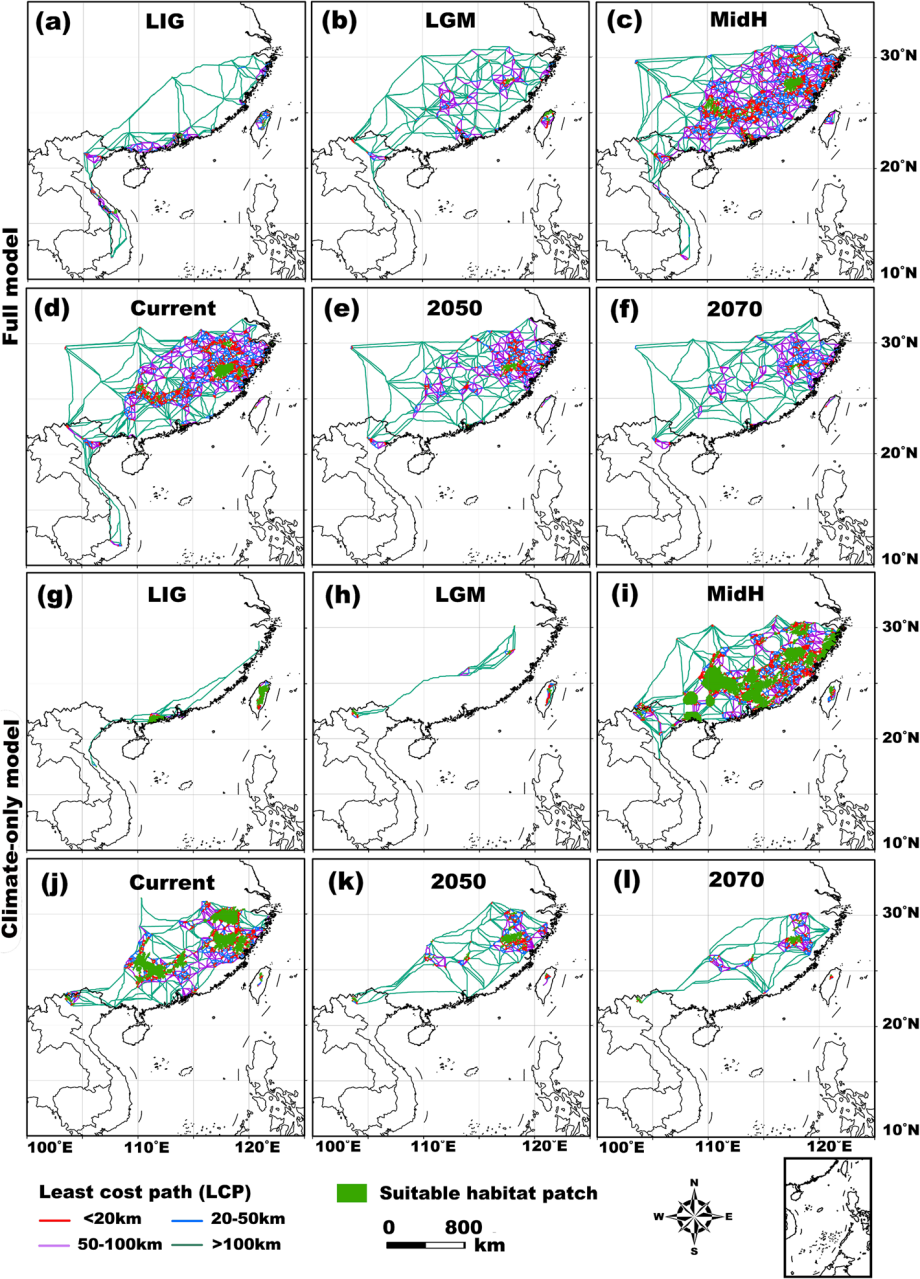
Suitable habitat patches and pairwise patch-to-patch least cost paths (LCPs) of *Quasipaa spinosa*. Top two rows: outputs are from the full model (all 22 environmental variables are included). Bottom two rows: outputs are based on the climate-only model (only the 14 bioclimatic variables are included)

Our results showed high landscape connectivity in the MidH and current time frames compared to those of the past (the LIG and LGM) and future (2050 and 2070; Fig. 5). The LCP values among habitat patches generally decreased across the ice ages (from the LIG to LGM) and increased after glaciations (from the LGM to the MidH and the current period) and then decreased again by more than half by 2050 and by nearly 75% by 2070 (Table 2). Accordingly, the mean LCP length and movement cost among patches revealed an increase-decrease-increase pattern over time (Table 2). This finding was robust to assumptions about maximum LCP lengths.

**Table 2:** The numbers, mean lengths, and mean movement costs of patch-to-patch LCPs (least cost paths) for *Quasipaa spinosa* over time under different LCP length limits based on the full and climate-only models

LCP length limit	20 km			50 km			100 km			No limit		
LCP	No.	Mean length (km)	Mean movement cost (10^5^)	No.	Mean length (km)	Mean movement cost (10^5^)	No.	Mean length (km)	Mean movement cost (10^5^)	No.	Mean length (km)	Mean movement cost (10^5^)
**Full model**												
LIG	11	11.0 ± 6.8	1.4 ± 3.8	28	31.1 ± 13.3	14.0 ± 11.9	41	48.6 ± 34.8	20.7 ± 15.9	85	290.4 ± 410.4	109.2 ± 143.9
LGM	9	15.1 ± 5.6	4.4 ± 2.1	19	27.5 ± 15.0	8.0 ± 4.5	21	31.7 ± 19.5	9.3 ± 6.0	33	162.7 ± 213.7	75.9 ± 103.6
MidH	161	12.7 ± 7.4	2.9 ± 2.4	276	23.4 ± 16.0	4.6 ± 3.9	329	33.0 ± 27.5	6.4 ± 5.9	420	102.4 ± 191.5	27.0 ± 60.5
Current	164	11.1 ± 8.5	1.8 ± 1.7	345	24.2 ± 18.5	4.6 ± 4.8	457	37.7 ± 30.0	6.8 ± 6.8	586	102.7 ± 199.1	26.7 ± 68.7
2050	46	13.3 ± 6.4	3.3 ± 3.5	91	29.7 ± 22.6	6.6 ± 6.4	117	42.6 ± 34.0	8.9 ± 8.4	158	134.2 ± 212.0	30.8 ± 52.9
2070	11	11.3 ± 5.0	1.8 ± 1.6	22	26.5 ± 18.9	5.9 ± 8.9	29	42.8 ± 36.8	8.6 ± 9.6	62	244.8 ± 263.6	43.4 ± 50.6
**Climate-only model**												
LIG	21	10.9 ± 9.3	6.0 ± 6.4	28	21.8 ± 24.1	11.4 ± 12.4	32	33.1 ± 39.7	14.2 ± 15.1	47	288.9 ± 433.9	82.2 ± 116.3
LGM	14	11.7 ± 6.1	2.1 ± 9.8	22	20.2 ± 13.7	3.7 ± 2.6	22	20.2 ± 13.7	3.7 ± 2.6	33	301.9 ± 551.1	77.1 ± 128.8
MidH	156	10.9 ± 6.2	3.2 ± 4.8	255	21.0 ± 15.4	5.0 ± 7.6	293	29.1 ± 26.1	6.2 ± 9.8	401	112.4 ± 207.2	24.5 ± 52.7
Current	83	10.0 ± 6.4	1.9 ± 3.3	131	20.7 ± 17.5	3.5 ± 5.9	170	34.1 ± 30.6	4.8 ± 7.6	250	135.3 ± 217.9	23.8 ± 49.6
2050	54	14.1 ± 10.0	5.8 ± 6.5	88	25.6 ± 19.2	8.5 ± 9.7	105	34.6 ± 28.2	9.9 ± 11.6	155	157.0 ± 263.1	37.6 ± 67.2
2070	184	11.6 ± 7.8	2.3 ± 4.0	277	19.7 ± 14.4	4.4 ± 7.9	318	26.8 ± 23.6	5.5 ± 9.9	392	86.9 ± 175.3	12.2 ± 23.6

The mean lengths and the mean movement costs are shown as the mean ± SD

## Discussion

Our results predicted how the locations and extents of suitable habitats for *Q. spinosa* varied and will vary as functions of past and future climate changes. The most striking finding is that the inferred future loss of habitat connectivity could lead to a decrease in individual dispersal, increasing the extinction risk of this threatened frog species. In summary, this study presents robust, spatially explicit predictions of historical and future suitable habitats for *Q. spinosa*.

Global paleoclimate fluctuations constitute dramatic cycles of environmental change on Earth. These fluctuations have profoundly shifted the distributions of many plant and animal species [60, 61]. Characterized by at least 30 intermittent glacial-interglacial cycles, the Pleistocene climatic oscillations are believed to have caused many iterations of massive species extirpations over large portions of their ranges, dispersal events to new locations, the trapping of species in refugia, and postglacial expansions [60, 61]. In East Asia, the climate is characterized as a unique monsoon system with dry winters and abundant precipitation in summers thanks to the uplift of the Qinghai-Tibetan Plateau. From the LIG to the MidH, East Asia experienced expanded ice caps and extensive glacier valley systems rather than extensive ice sheets, especially during the LGM [60, 62–64]. Such relatively moderate conditions can be conducive for species to retain hospitable habitats or to make relatively easy migrations to newly suitable areas [65, 66]. Previous studies suggested that during cold periods, some amphibian species in southern and eastern China might disperse through streams that provide connectivity to refugia, including some mountainous regions, which can be topographically heterogeneous with abundant suitable habitats with relatively stable microclimates [67, 68]. We found that there was a north-easterly shift of suitable *Q. spinosa* habitats from the LIG to the LGM as a result of warming conditions and the angle of the coastline with the South China Sea*.* This shift may have moved frog populations closer to mountains that could function as refugia over geologic time, with the frogs using the abundant streams and river branches within the water systems (including the Yangtze River network and the Pearl River network) in South China as their dispersal passages [69]. The suitable habitats generally moved from the regions around the southern coasts of China and the eastern countries of south-eastern Asia to some mountainous areas in eastern China, including Huangshan, Dabieshan-Wuyishan-Luoxiaoshan, Nanling, Wushan-Xuefengshan, and the Qinling-Dabashan Mountains (Fig. 2). As the temperatures rose after glaciation (in the MidH and the current period), suitable habitats were greatly enlarged and expanded into surrounding regions. In the coming decades, the continuously changing climate will contract and restrict the potential distribution of the frogs around the hilly areas of eastern China (Fig. 2).

We found that precipitation in the dry season, together with temperature variables and rainfall abundance in eastern Asia, acted as the main drivers of the dynamics of suitable habitats (Fig. S1 in Additional File 2). We found that not only did the cold and dry climatic conditions that occurred during the ice ages pose challenges for the survival of *Q. spinosa*, but also that the “overwarm” climate in the future could be a threat to these environmentally sensitive and non-heat-tolerant frogs. This phenomenon has also been found in other amphibians [61, 67, 69]. The dual climate challenges mean that the present conditions are optimal for *Q. spinosa* when compared with all the time slices considered. Our findings suggest that hilly areas around the lower reaches of the Yangtze River, eastern parts of the Yunnan-Guizhou Plateau (also named the Yunnan-Kweichow Plateau), the south-eastern mountains in China, and the Sino-Vietnamese transboundary areas in northern Vietnam (Fig. 2) are high-priority areas for protection. Therefore, on-going and future protection actions should emphasize landscape connectivity concurrently with habitat area and the number of habitat patches.

Our results showed that landscape connectivity was low during the cold periods in the geologic past (from the LIG to the LGM), followed by dramatic improvements post-glaciation. However, we also found that connectivity is likely to decline again in the face of additional warming. Considering the dispersal capacity of anurans [54], our projections of connectivity based on the assumption of a 20-km path-length limit could be the closest to reality and could provide valuable references to conservation planning for both the present and the future [70]. Regardless of the exact maximum length of dispersal, frogs may not use these paths due to the limited mobility and strict physiological constraints of anurans. As a result, additional research is needed to more fully inform the population management of *Q. spinosa* [15, 71]. Even assuming we correctly identified inter-patch passageways with the highest potential and permeability for individuals to disperse successfully, constraints to animal movements are complex, with a number of stochastic ecological and landscape factors that may have important effects [15, 19]. Hence, other possible paths that could promote individual exchanges among populations or patches should also be seriously considered in protection programmes. Small isolated fragments of suitable habitats, which serve as “stepping stones” between large patches and dispersal stopovers for frogs, cannot be ignored [cf. 71]. In addition, to reduce possible edge effects and anthropogenic influences, buffering the LCPs into width-sufficient patch-to-patch corridors based on the species’ home range requirements could be a critical step, and the functions or effectiveness of these buffers should be tested with population monitoring data and empirical gene flow studies among patches [14, 15].

Given the threats that already exist [20, 22, 24] and those that will most likely emerge under climate change and due to other anthropogenic impacts in the future, we suggest that appropriate protection strategies and strengthened conservation efforts should be developed immediately and be modified as habitat degradation and fragmentation continue. Specifically, based on the results of this study, we propose the following conservation recommendations for *Q. spinosa*. As the first step towards efficient protection and population sustainability actions [72], several representative areas and critical refugia (i.e., habitats, patches, or local populations) identified here should be focused on as key suitable habitats, sources of genetic diversity, and conservation priorities. These areas include the hilly and mountainous regions in southern, south-eastern, and south-western China and the transboundary region between China and Vietnam, which requires transboundary cooperation for species protection. Second, the LCPs in our map of the present and future distributions of *Q. spinosa* habitats should be emphasized in conservation planning. Actions that enhance inter-patch dispersal and the exchange of frog individuals between patches should be considered. For example, preserving LCPs as sufficiently wide ecological connections and preserving habitat stability within corridors should be pursued as conservation priorities. Because of future reductions in landscape connectivity, long-term resources should be allocated to monitor and analyse habitat quality, individual dispersal, and gene flow among patches to assess conservation effectiveness. Third, the networks of protected areas within the extant range of *Q. spinosa* should be reassessed in light of our findings. As the coverage ratios of habitat areas and patch numbers in the protected area network are relatively low (< 30% or approximately 30%), selecting new protected areas and adjusting existing protected areas may be urgent in order to ensure that sufficient habitat areas are protected for the species, especially in the eastern part of its distribution region. Finally, to help counteract population decline, environmental education in local communities, the refinement of protection laws and management systems, and the restriction of human activities may also be necessary.

We recognize that some caveats exist in this study. Our results only predict and quantify the dynamics of suitable habitats and landscape connectivity at the landscape scale but do not consider the potential impacts of climate change at a finer resolution. Second, more efforts, such as analysing gene flow, monitoring individuals’ dispersals among populations, and comparing different landscape models, are needed to empirically validate some of the assumptions made in our model. The third caveat is that the influences of some other ecological factors, such as predation risk and local conservation efforts, should be considered to explain local population dynamics [14, 73]. Furthermore, if we took into account the human impacts on and microhabitat requirements of *Q. spinosa* when modelling habitat suitability, the potentially suitable habitats would be more fragmented (Fig. 2; Table 1). An additional problem is the assumption of the constancy of the non-climatic factors (i.e., AI, PET, Landcover, NPP, Biome, HFP) over time; that is, we use current data as conservative predictions of these variables for the future and past. This could lead to a caveat in our modelling results, especially those from the full models (some of the non-climatic variables showed relatively high contributions to habitat suitability). However, we considered the method used in this study to be a reasonable way to restrict additional uncertainty, as these variables could be influenced by a variety of factors (not only climatic but also anthropogenic-mediated factors, etc.) and are thus difficult to estimate, and timely analyses need to be conducted when updated data are available [37]. Finally, to better capture the consequences of future climate change, i.e., range shifts, it would be necessary to quantify genetic diversity and associated ecological drivers of geographic populations [74]. Despite these caveats, by exploring shifts in the suitable habitat and landscape connectivity of an anuran species that occur as a consequence of climate change, our findings can serve as a template for conservation planning for a variety of amphibians that face the interlocking threats of habitat fragmentation and climate change.

## Conclusions

Our results reveal that suitable habitats of *Q. spinosa* obviously declined and shifted northwardly during the Last Glacial Maximum and expanded after the ice age. In the future, suitable habitats are projected to decrease again due to “overwarm” climatic conditions. The dynamics of suitable habitats also lead to habitat connectivity changes in this species during post-glaciation periods. In the face of future climate change, an increase in habitat fragmentation and a decrease in habitat connectivity are predicted. We propose that the mountainous areas in the southern China and Sino-Vietnamese transboundary regions can be considered conservation priorities for this frog species. Given that amphibians are highly sensitive to environmental changes, this study highlights that the dynamics of environmental suitability and habitat connectivity should be considered to guide large-scale conservation management measures for endangered amphibian species under climate change.

## Supplementary Information

**Additional file 1: Table S1.** Environmental variables used in potential distribution modelling for *Quasipaa spinosa*. **Table S2.** Habitat quality score and movement cost score of each environmental variable and their weights in movement cost surface for *Quasipaa spinosa*.

**Additional file 2: Figure S1.** Jackknife analyses on the contributions of environmental variables when modelling potential distributions for *Quasipaa spinosa*: (a) full model (all 22 environmental variables are included), (b) climatic-only model (only 14 bioclimatic variables are included).

## Data Availability

The datasets used and/or analysed during the current study are available from the corresponding author on reasonable request.
