# Supplementary material for: Combining the responses of habitat suitability and connectivity to climate change for an East Asian endemic frog
Source: Front Zool. 2021 Mar 26;18:14. doi: 10.1186/s12983-021-00398-w (PMC7995727; doi:10.1186/s12983-021-00398-w)
Supplement: Supplementary file 1 — Additional file 1: Table S1. Environmental variables used in potential distribution modelling for Quasipaa spinosa. Table S2. Habitat quality score and movement cost score of each environmental variable and their weights in movement cost surface for Quasipaa spinosa. [file 12983_2021_398_MOESM1_ESM.docx]

**Table S1.** Environmental variables used in potential distribution modelling for *Quasipaa spinosa*.

| Variable group | Environmental variable | Abbreviation | Data source |
| --- | --- | --- | --- |
| Climate | Annual mean temperature (°C) | Bio1 | WorldClim 1.4, http://www.worldclim.org/ |
|  | Mean temperature diurnal range (°C) | Bio2 | WorldClim 1.4, http://www.worldclim.org/ |
|  | Temperature seasonality (°C) | Bio4 | WorldClim 1.4, http://www.worldclim.org/ |
|  | Maximum temperature of the warmest month(°C) | Bio5 | WorldClim 1.4, http://www.worldclim.org/ |
|  | Minimum temperature of the coldest month (°C) | Bio6 | WorldClim 1.4, http://www.worldclim.org/ |
|  | Temperature annual range (°C) | Bio7 | WorldClim 1.4, http://www.worldclim.org/ |
|  | Mean temperature of the warmest quarter (°C) | Bio10 | WorldClim 1.4, http://www.worldclim.org/ |
|  | Mean temperature of the coldest quarter (°C) | Bio11 | WorldClim 1.4, http://www.worldclim.org/ |
|  | Annual precipitation (mm) | Bio12 | WorldClim 1.4, http://www.worldclim.org/ |
|  | Precipitation of the wettest month (mm) | Bio13 | WorldClim 1.4, http://www.worldclim.org/ |
|  | Precipitation of the driest month (mm) | Bio14 | WorldClim 1.4, http://www.worldclim.org/ |
|  | Precipitation seasonality (mm) | Bio15 | WorldClim 1.4, http://www.worldclim.org/ |
|  | Precipitation of the wettest quarter (mm) | Bio16 | WorldClim 1.4, http://www.worldclim.org/ |
|  | Precipitation of the driest quarter (mm) | Bio17 | WorldClim 1.4, http://www.worldclim.org/ |
| Habitat | Aridity index | AI | CGIAR-CSI Global-Aridity and Global-PET Database, http://www.cgiar-csi.org/data/global-aridity-and-pet-database/ |
|  | Annual potential evapo-transpiration (mm) | PET | CGIAR-CSI Global-Aridity and Global-PET Database, http://www.cgiar-csi.org/data/global-aridity-and-pet-database/ |
|  | Landcover type | Landcover | Global Landcover 2000, http://ies.jrc.ec.europa.eu/global-land-cover-2000/ |
|  | Net primary productivity (g carbon/m^2^/year) | NPP | Thematic Database for Human–Earth System,http://www.data.ac.cn/ |
|  | Percentage of water area (%) | Water area | GPWv4: Land and Water Area, http://dx.doi.org/10.7927/H45M63M9/ |
| Biogeography | Terrestrial ecoregion | Biome | Terrestrial Ecoregions of the World, https://www.worldwildlife.org/publications/terrestrial-ecoregions-of-the-world/ |
| Topography | Slope (°) | Slope | Calculated based onDEM datafrom CGIAR-CSI, http://srtm.csi.cgiar.org/ |
| Human impact | Human footprint index | HFP | Last of the Wild DataVersion 2, http://sedac.ciesin.columbia.edu/wildareas/ |

**Table S2.** Habitat quality score and movement cost score of each environmental variable and their weights in movement cost surface for *Quasipaa spinosa*.

| Movement cost score | Habitat suitability score | Bio1 (°C) | Bio2 (°C) | Bio4 (°C) | Bio5 (°C) | Bio6 (°C) | Bio7 (°C) | Bio10 (°C) | Bio11 (°C) | Bio12 (mm) | Bio13 (mm) | Bio14 (mm) |
| --- | --- | --- | --- | --- | --- | --- | --- | --- | --- | --- | --- | --- |
| 10 | 1 | >27.5 | >14 | >100 | <10 | <-15 | <10 | <5 | >25 | <500 | <100 | <10 |
| 9 | 2 | <0 | 12-14 | <20 | 10-15 | >20 | >40 | >31 | <-10 | 500-1000 | >1000 | 10-20 |
| 8 | 3 | 25-27.5 | 11-12 | 20-30 | 15-17.5 | −15-10 | 10-15 | 5-10 | −10-5 | 1000-1250 | 100-200 | 20-30 |
| 7 | 4 | 0-5 | 10-11 | 90-100 | >35 | 15-20 | 32.5-40 | 10-15 | 20-25 | 2500-4000 | 800-1000 | >180 |
| 6 | 5 | 5-10 | 9-10 | 30-40 | 17.5-20 | −10-5 | 15-17.5 | 29-31 | −5-0 | >4000 | 600-800 | 140-180 |
| 5 | 6 | 10-12.5 | 8-9 | 40-50 | 32.5-35 | 10-15 | 17.5-20 | 15-20 | 17.5-20 | 2400-2500 | 400-600 | 100-140 |
| 4 | 7 | 20-25 | <5 | 80-90 | 20-22.5 | −5-2.5 | 20-25 | 28-29 | 0-4 | 2200-2400 | 200-225 | 60-100 |
| 3 | 8 | 15-17.5 | 6-7 | 50-60 | 22.5-25 | 5-10 | 25-27.5 | 20-22.5 | 15-17.5 | 1250-1500 | 350-400 | 50-60 |
| 2 | 9 | 12.5-15 | 5-6 | 70-80 | 30-32.5 | 2.5-5 | 30-32.5 | 25-28 | 10-15 | 1500-2000 | 225-250 | 30-40 |
| 1 | 10 | 17.5-20 | 7-8 | 60-70 | 25-30 | −2.5-2.5 | 27.5-30 | 22.5-25 | 4-10 | 2000-2200 | 250-350 | 40-50 |
| Weight (all variables) | | 0.2568 | 0.3638 | 0.316 | 0.0965 | 0.3412 | 0.2845 | 0.0982 | 0.3334 | 0.4966 | 0.2932 | 0.7431 |
| Weight (only bioclimatic variables) | | 0.2638 | 0.3675 | 0.3166 | 0.0999 | 0.3552 | 0.2937 | 0.1012 | 0.3481 | 0.4965 | 0.2851 | 0.7357 |

**Table S2.** Habitat quality score and movement cost score of each environmental variable and their weights in movement cost surface for *Quasipaa spinosa* (continued).

| Movement cost score | Habitat suitability score | Bio15 (mm) | Bio16 (mm) | Bio17 (mm) | PET (mm) | AI | Landcover | NPP (g carbon/m^2^/year) | Water area (%) | Slope (°) | Biome | HFP |
| --- | --- | --- | --- | --- | --- | --- | --- | --- | --- | --- | --- | --- |
| 10 | 1 | >110 | <250 | <10 | >1600 | <10000 | Deciduous broadleaf forest | >3000 | >70 | 14-20 | Open water | <10 |
| 9 | 2 | 90-110 | 250-500 | 10-40 | 1450-1600 | 10000-11000 | Cropland | <300 | 60-70 | 20-25 | Farmland | 10-20 |
| 8 | 3 | 80-90 | >2500 | 40-60 | <800 | 11000-12000 | Woodland, grassland | 2500-3000 | 50-60 | 25-30 | Northern Indochina subtropical forest | 35-40 |
| 7 | 4 | 65-80 | 1100-2500 | 60-80 | 1400-1450 | 12000-13000 | Evergreen broadleaf forest | 2000-2500 | 40-50 | 30-35 | Admiralty Islands lowland rain forest | 20-35 |
| 6 | 5 | <20 | 500-600 | 80-100 | 800-900 | 13000-15000 | Closed shrubland,  open shrubland, bare ground | 300-800 | 30-40 | 35-37 | Northern Annamites rain forest | 40-45 |
| 5 | 6 | 20-30 | 1000-1100 | >500 | 1300-1400 | 15000-17000 | Wooded grassland | 1800-2000 | <2 | >37 | South China-Vietnam subtropical evergreen forest | 45-60 |
| 4 | 7 | 30-40 | 600-700 | 200-500 | 1200-1300 | 23000-40000 | Mixed forest | 1600-1800 | 20-30 | <5 | Changjiang Plain evergreen forest | 60-70 |
| 3 | 8 | 40-45 | 800-900 | 100-125 | 1050-1150 | >40000 | Water | 1400-1600 | 10-20 | 13-14 | Southern Annamites montane rain forest | 70-80 |
| 2 | 9 | 55-65 | 700-800 | 125-150 | 1150-1200 | 17000-20000 | Evergreen needleleaf forest | 1200-1400 | 5-10 | 5-10 | Orinoco Delta swamp forest | 80-90 |
| 1 | 10 | 45-55 | 900-1000 | 150-200 | 900-1050 | 20000-23000 | Urban and built | 800-1200 | 2-5 | 10-13 | Jian Nan subtropical evergreen forest | >90 |
| Weight (all variables) | | 0.4392 | 0.3493 | 0.7178 | 0.1985 | 0.5697 | 0.1655 | 0.2981 | 0.0035 | 0.1092 | 0.6987 | 0.0467 |
| Weight (only bioclimatic variables) | | 0.4379 | 0.3456 | 0.7084 |  |  |  |  |  |  |  |  |
