# Supplementary material for: Combining the responses of habitat suitability and connectivity to climate change for an East Asian endemic frog
Source: Front Zool. 2021 Mar 26;18:14. doi: 10.1186/s12983-021-00398-w (PMC7995727; doi:10.1186/s12983-021-00398-w)
Supplement: Supplementary file 2 — Additional file 2: Figure S1. Jackknife analyses on the contributions of environmental variables when modelling potential distributions for Quasipaa spinosa: (a) full model (all 22 environmental variables are included), (b) climatic-only model (only 14 bioclimatic variables are included). [file 12983_2021_398_MOESM2_ESM.docx]

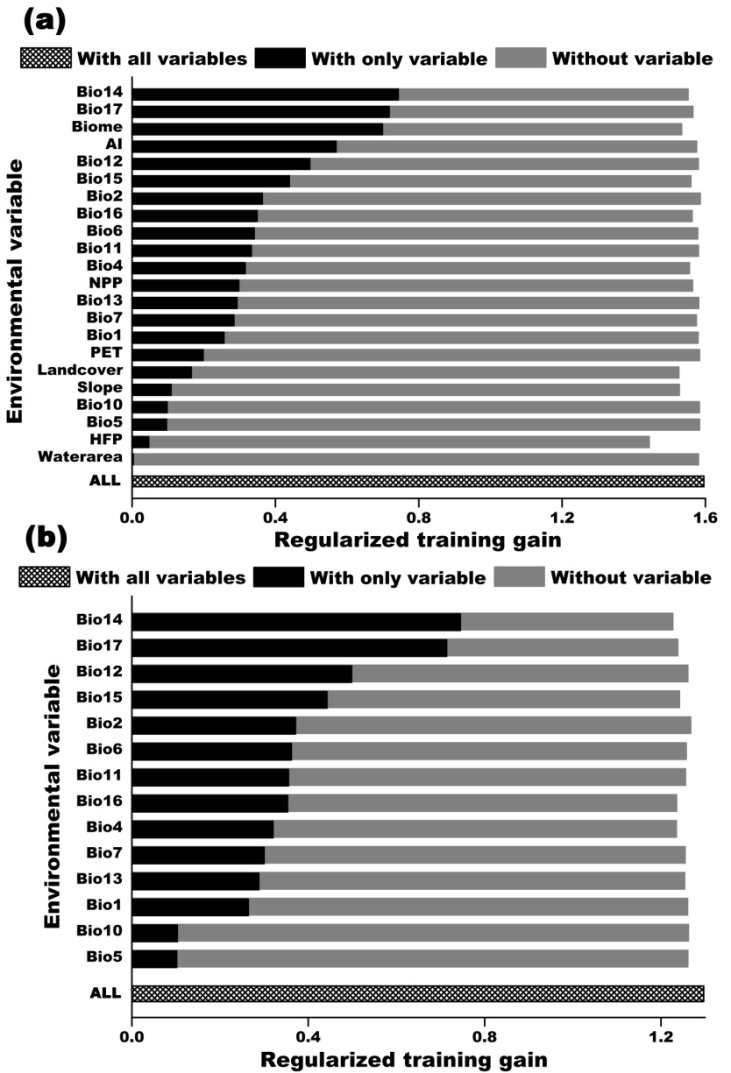


**Figure S1.** Jackknife analyses on the contributions of environmental variables when modelling potential distributions for *Quasipaa spinosa*: (a) full model (all 22 environmental variables are included), (b) climate-only model (only 14 bioclimatic variables are included).
